# ANCA-negative eosinophilic granulomatosis with polyangiitis diagnosed via external auditory canal biopsy: A case report

**DOI:** 10.1016/j.rmcr.2026.102528

**Published:** 2026-10-23

**Authors:** Riao Dao, Yunsiriguleng Wu, Muqiri Xiang, Meijing Zhang, Zhihong Wang, Qiao Yu, MaoLu Ri, A Latangaridi

**Affiliations:** a Department of Psychosomatic Medicine, International Mongolian Medical Hospital of Inner Mongolia Autonomous Region, Hohhot, 010010, PR China; b Intensive Care Unit, International Mongolian Medical Hospital of Inner Mongolia Autonomous Region, Hohhot, 010010, PR China

**Keywords:** Eosinophilic granulomatosis with polyangiitis, Eosinophilia, ANCA-Negative vasculitis, External auditory canal, Ear manifestations

## Abstract

**Background:**

Eosinophilic granulomatosis with polyangiitis (EGPA) is an uncommon systemic vasculitis characterized by asthma, eosinophilia, and multisystem involvement. The diagnosis of ANCA-negative EGPA may be challenging because serological findings are often non-specific, making histopathological confirmation important. We report a case of ANCA-negative EGPA diagnosed by biopsy of the external auditory canal, an uncommon but readily accessible site of disease involvement.

**Case presentation:**

A 22-year-old Mongolian woman presented with progressive chest tightness and dyspnea. Her medical history was notable for asthma and nasal polyps. She also reported bilateral ear fullness and tinnitus. Laboratory investigations demonstrated marked eosinophilia (35.8% of leukocytes), while ANCA testing was negative. Otoscopic examination revealed inflammatory changes in the left external auditory canal, and biopsy was subsequently performed. Histopathological examination showed vasculitis with prominent eosinophilic infiltration, supporting the diagnosis of EGPA. Treatment with systemic corticosteroids resulted in rapid clinical improvement. At three-month follow-up, eosinophil counts had normalized and no evidence of disease progression was observed.

**Conclusions:**

This case highlights the potential diagnostic value of external auditory canal biopsy in patients with suspected ANCA-negative EGPA. Otologic manifestations may provide accessible sites for tissue sampling when histological confirmation is required. Careful otologic examination should therefore be considered during the evaluation of patients with suspected EGPA.

## 1 Introduction

Eosinophilic granulomatosis with polyangiitis (EGPA), formerly known as Churg-Strauss syndrome, is a systemic vasculitis affecting small and medium-sized blood vessels. The condition typically presents with asthma, elevated eosinophils in the blood, and various organ manifestations [1]. Many patients also have allergic conditions like rhinitis or nasal polyps before the vasculitis develops. Definitive diagnosis usually requires tissue confirmation, as no single test can confirm the disease on its own.

EGPA is uncommon, with an estimated incidence of about 1 to 2 cases per million people per year. The average age at onset is around 44 years, and men are affected slightly more often than women. Roughly 30-40% of patients test positive for ANCA, most commonly MPO-ANCA [2]. The remaining 60-70% are ANCA-negative, which makes diagnosis more challenging since serology alone cannot confirm the disease [3].

Ear problems occur relatively frequently in EGPA. Studies report otologic involvement in 19-61% of patients [4], [5], including middle-ear effusion and hearing loss [6]. However, these studies primarily describe middle-ear and audiovestibular disease rather than diagnostic biopsy of the external auditory canal. We present this case because an actively inflamed external auditory canal provided a readily accessible site for tissue confirmation.

## 2 Case presentation

### 2.1 Patient information

A 22-year-old Mongolian woman was referred to our hospital after repeated asthma-related hospitalizations at several hospitals in Mongolia. Asthma had been diagnosed approximately two years earlier, and she had persistent nasal discomfort and nasal polyps. During the two weeks before admission, chest tightness, dyspnea, and cough with yellow sputum worsened. She also reported bilateral ear fullness and tinnitus, more prominent on the left, together with subjective numbness of the left arm and right side of the tongue. No nerve-conduction study or electromyography was performed. These symptoms were therefore recorded as subjective sensory complaints rather than objectively confirmed neuropathy. She had no history of smoking, serious infection, or relevant family history.

### 2.2 Clinical findings

On physical examination, bilateral wheezing was present. Because the patient had nasal and nasopharyngeal discomfort with bleeding, she requested same-day otorhinolaryngology assessment. Otorhinolaryngologic examination showed allergic rhinitis, sinusitis, nasal polyps, chronic otitis media, and bilateral erythematous polypoid soft-tissue lesions adjacent to the tympanic membranes; inflammation of the left external auditory canal was more prominent (Fig. 1A1-A2). Chest CT demonstrated bilateral pulmonary infiltrates, initially interpreted as infection, with mild pleural thickening. Their subsequent resolution with corticosteroid therapy supported eosinophilic pulmonary involvement rather than infection.

**Fig. 1:**
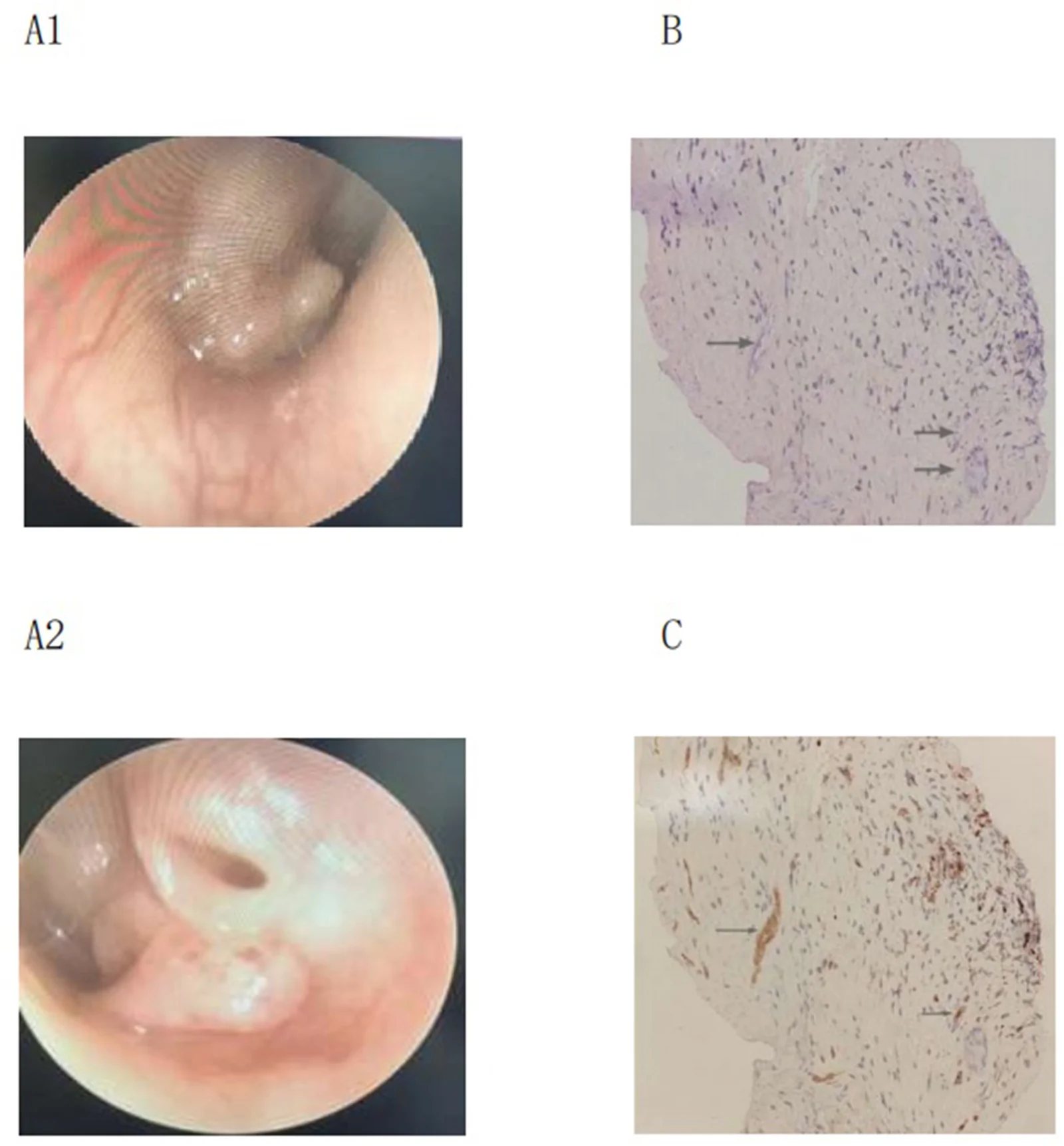
Otoscopic and histopathological findings. (A1) Otoscopic view showing edematous narrowing of the external auditory canal and a dull, thickened tympanic membrane. (A2) Otoscopic view showing more prominent canal erythema and a focal erythematous polypoid soft-tissue lesion adjacent to the tympanic membrane. (B) H&E-stained section showing vessel-wall necrosis with eosinophilic and other inflammatory-cell infiltration (arrows). (C) CD31 immunohistochemistry highlighting endothelial cells in the involved vessels; CD31 was used to identify vascular structures, not as an independent marker of vasculitis. Scale bar = 100 μm.

### 2.3 Diagnostic assessment

Her blood work was striking: eosinophils made up 35.8% of her white blood cells, giving an absolute count of 6.5 × 10^9^/L (see Table 1). Her IgE level was very high at over 1000 IU/mL. Kidney function and other basic labs were normal.

**Table 1:** Laboratory findings at admission.

Parameter	Patient Value	Reference Range
**Complete blood count**		
White blood cell count (×10^9^/L)	18.2	3.5-9.5
Eosinophil percentage (%)	35.8	0.4-8.0
Absolute eosinophil count (×10^9^/L)	6.5	0.02-0.52
**Serum biochemistry**		
Serum creatinine (μmol/L)	31.8	41.0-81.0
Albumin (g/L)	36.9	40-55
Lactate dehydrogenase (U/L)	264.5	120-250
C-reactive protein (mg/L)	35.49	<10
**Immunological tests**		
c-ANCA	Negative	Negative
Anti-PR3 antibodies	Negative	Negative
Total serum IgE (IU/mL)	>1000	0-100

We ordered a full ANCA panel, testing for both PR3-ANCA and MPO-ANCA. This is standard practice when evaluating suspected ANCA-associated vasculitis [7]. Both assays were negative, a finding that is common in EGPA [3].

The combination of asthma, nasal polyps, marked eosinophilia, pulmonary infiltrates, and sensory complaints raised strong suspicion for EGPA. Although she fulfilled several clinical classification items, tissue confirmation was sought because ANCA testing was negative and there was no prior evidence of eosinophilic extravascular tissue infiltration. After multidisciplinary discussion, our hospital team recommended biopsy to document eosinophilic infiltration and vessel-wall injury. Nasal polyps were present but were not the actively symptomatic or most readily accessible lesion at that visit; the inflamed external auditory canal was the accessible site requested and examined by otorhinolaryngology, and was therefore selected. Written consent was obtained for biopsy and publication.

## 3 Results

### 3.1 Histopathological findings

The biopsy from the left external auditory canal showed fibrous tissue containing small blood vessels with vessel-wall necrosis and inflammatory infiltration by neutrophils, lymphocytes, and prominent eosinophils (Fig. 1B). Eosinophils were present within and around the vessel walls. CD31 immunostaining highlighted endothelial cells and confirmed that the examined structures were blood vessels (Fig. 1C). CD31 was not interpreted as an independent marker of vasculitis; the diagnosis rested on the H&E evidence of vessel-centered eosinophilic inflammation, fibrinoid necrosis, and vessel-wall injury.

### 3.2 Final diagnosis

Integrating the clinical and pathological findings, we diagnosed ANCA-negative EGPA. After mimicking conditions had been excluded, the patient met the 2022 ACR/EULAR classification criteria for EGPA, with obstructive airway disease (+3), nasal polyps (+3), and a maximum blood eosinophil count of at least 1 × 10^9^/L (+5), yielding a total score of 11, above the required threshold of 6 [8]. The reported left-arm and right-sided tongue numbness were subjective sensory symptoms that improved with treatment; because no nerve-conduction study or electromyography was performed, they were not scored as objectively confirmed mononeuritis multiplex. Histopathology provided additional diagnostic support by demonstrating vessel-centered eosinophilic inflammation and vessel-wall injury.

## 4 Treatment and outcome

We started her on prednisone at 1 mg/kg body weight daily. Within a week, her breathing felt much better and the chest tightness had eased. The numbness in her arm and tongue gradually improved over the following two weeks. Her ear symptoms also improved. We tapered the steroid dose slowly over six weeks based on her clinical response and eosinophil levels. Rheumatology consultation was obtained; mepolizumab was discussed but deferred because of the rapid response and resource considerations, with biologic therapy to be reconsidered if relapse occurs [9].

At her three-month follow-up visit, she felt completely well. All her symptoms—respiratory, neurological, and ear-related—had resolved. Her eosinophil count was normal at 0.3 × 10^9^/L, and a repeat chest CT showed the lung infiltrates had cleared. She has had no signs of disease recurrence so far.

## 5 Discussion

Ear involvement is a recognized manifestation of EGPA, although it may be overlooked in routine clinical practice. Previous studies have reported otologic manifestations in approximately 19-61% of patients [4], [5], and persistent middle-ear effusion may contribute to permanent hearing loss [6]. These manifestations are thought to result from eosinophilic inflammation and vasculitic involvement of otologic structures. In selected patients, careful examination may also identify an accessible inflammatory lesion in the external auditory canal.

The diagnosis of ANCA-negative EGPA remains challenging because serological testing alone is insufficient for confirmation. In such cases, diagnosis relies on the integration of clinical manifestations, laboratory findings, exclusion of mimics, and, when feasible, histopathological evidence [3], [8]. The 2022 ACR/EULAR criteria are classification rather than diagnostic criteria; nevertheless, they provide a current standardized framework after a diagnosis of small- or medium-vessel vasculitis has been established and mimics have been excluded [8]. Identification of an accessible biopsy site may therefore be clinically valuable.

To assess the novelty of the biopsy site, we searched PubMed using combinations of “eosinophilic granulomatosis with polyangiitis” or “Churg-Strauss” with “external auditory canal,” “ear canal,” and “biopsy” (search updated September 29, 2026). We also reviewed a systematic review and representative cohort studies of otologic EGPA [4], [5], [6]. These sources describe middle-ear, inner-ear, and audiovestibular manifestations but did not identify a clearly documented case in which external-auditory-canal biopsy established the diagnosis. Thus, to our knowledge, this is the first clearly documented report of tissue-supported EGPA diagnosed from an external-auditory-canal lesion. When such a visible inflammatory lesion is present, biopsy offers procedural accessibility and the potential to obtain diagnostically relevant tissue. In this patient, H&E examination demonstrated fibrinoid necrosis, vessel-wall injury, and vessel-centered eosinophilic infiltration. CD31 only highlighted the endothelial lining and was not used independently to establish vasculitis.

With regard to ANCA testing, MPO-ANCA is the most frequently detected ANCA subtype in EGPA when ANCA positivity is present. Current recommendations support antigen-specific testing for both PR3-ANCA and MPO-ANCA when ANCA-associated vasculitis is suspected [7]. In our patient, both assays were negative, supporting classification as ANCA-negative EGPA.

The patient responded favorably to systemic corticosteroid therapy. This outcome is consistent with previous reports indicating that glucocorticoid treatment may be effective in patients without severe cardiac, renal, or gastrointestinal involvement [10]. The absence of major organ-threatening manifestations likely contributed to the favorable clinical course observed in this case. Mepolizumab is an evidence-based option for relapsing or refractory EGPA and was reserved for reconsideration if relapse occurred [9].

Several limitations should be acknowledged. First, this report describes a single patient, limiting the generalizability of the findings. Second, the three-month follow-up is short, and longer observation is required because relapse is common in EGPA [10]. In addition, biopsy of the external auditory canal is feasible only when a visible inflammatory lesion is present. Despite these limitations, this case suggests that otologic assessment may provide valuable diagnostic information in selected patients with suspected EGPA.

## 6 Conclusions

This case illustrates that examining the ears should be part of evaluating patients with possible EGPA. When the external ear canal shows visible inflammation, biopsying this area can provide the tissue diagnosis needed in ANCA-negative cases. Early recognition and treatment matter, as they can prevent serious complications. The ear canal is an underused option that may prove helpful in selected patients.

## CRediT authorship contribution statement

**Riao Dao:** Project administration, Conceptualization. **Yunsiriguleng Wu:** Visualization, Formal analysis, Data curation. **Muqiri Xiang:** Writing – original draft, Software. **Meijing Zhang:** Writing – original draft, Resources. **Zhihong Wang:** Methodology, Formal analysis. **Qiao Yu:** Formal analysis, Data curation. **MaoLu Ri:** Writing – review & editing, Supervision, Software. **A Latangaridi:** Investigation, Data curation.

## Ethics declaration

Written informed consent to take part in the study and to publish the article has been obtained from all participants or their legal representatives. The privacy rights of participants have been observed.

This study included organ or tissue donors. This study includes human biological material and consent was obtained by donors, or their next of kin or legal representatives, for use in this study and for publication of the article. The samples used in this research were not sourced from executed prisoners or prisoners of conscience.

This study was performed in compliance with relevant laws, regulatory frameworks and guidelines where the research took place. The Institutional Review Board of Inner Mongolia International Mongolian Medical Hospital granted an exemption. The authors provided the following explanation: This case report was exempt from formal ethics committee review based on the institutional guidelines of Inner Mongolia International Mongolian Medical Hospital, as it describes routine clinical care and does not constitute interventional research. The study was conducted in accordance with the Declaration of Helsinki and relevant Chinese ethical guidelines. Written informed consent for participation and publication, including accompanying images, was obtained from the patient.

This research follows the CARE guidelines and the CARE checklist.

## Ethics approval and consent to participate:

Under institutional policy, formal ethics committee approval was not required for this single-patient report of routine clinical care. The report was prepared in accordance with the Declaration of Helsinki and applicable Chinese ethical guidance.

## Consent for publication:

Written informed consent was obtained from the patient for publication of this case report and any accompanying images.

## Data availability

Due to patient privacy concerns, the data cannot be publicly shared. Requests for data access can be directed to the corresponding author.

## Ethics approval and consent to participate

Under institutional policy, formal ethics committee approval was not required for this single-patient report of routine clinical care. The case report was prepared in accordance with the Declaration of Helsinki and applicable Chinese ethical guidance.

## Consent for publication

Written informed consent was obtained from the patient for publication of this case report and the accompanying clinical and histopathological images.

## Availability of data and materials

The data supporting the findings of this case report are not publicly available because of patient privacy considerations. Reasonable requests for additional information may be directed to the corresponding author, subject to applicable ethical and privacy requirements.

## Funding

This work was supported by the National TCM Advantageous Specialty Construction Project of Inner Mongolia International Mongolian Medical Hospital.

## Declaration of competing interest

The authors declare that they have no known competing financial interests or personal relationships that could have appeared to influence the work reported in this manuscript.
