# Supplementary material for: ANCA-negative eosinophilic granulomatosis with polyangiitis diagnosed via external auditory canal biopsy: A case report
Source: Respir Med Case Rep. 2026 Oct 23;64:102528. doi: 10.1016/j.rmcr.2026.102528 (PMC13722062; doi:10.1016/j.rmcr.2026.102528)
Supplement: Supplementary file 1 [file mmc1.pdf]

CARE Checklist of information to include when writing a case report

Topic	Item	Checklist item description	Reported on Line
Title	1	The diagnosis or intervention of primary focus followed by the words "case report"	Page 1, Title
Key Words	2	2 to 5 key words that identify diagnoses or interventions in this case report, including "case report"	Page1, Keywords
Abstract (no references)	3a	Introduction: What is unique about this case and what does it add to the scientific literature?	Page1, Abstract-keywords
	3b	Main symptoms and/or important clinical findings	Page1, Abstract-Casrpresentation
	3c	The main diagnoses, therapeutic interventions, and outcomes	Page1, Abstract-Casepresentation
	3d	Conclusion—What is the main "take-away" lesson(s) from this case?	Page1, Abstract-Conclusions
Introduction	4	One or two paragraphs summarizing why this case is unique (**may include references**)	Page1-2, Introduction
Patient Information	5a	De-identified patient specific information	Page2, "A 22-year-old Mongolian Women"
	5b	Primary concerns and symptoms of the patient	Page2, Case presentation paragraph 1
	5c	Medical, family, and psycho-social history including relevant genetic information	Page2, Case presentation paragraph 1
	5d	Relevant past interventions with outcomes	Not applicable (no prior relevant interventions)
Clinical Findings	6	Describe significant physical examination (PE) and important clinical findings	Page 2, Case presentation (neurological symptoms: numbness of left upper limb and right side of tongur)
Timeline	7	Historical and current information from this episode of care organized as a timeline	NA (not included in this case report)
Diagnostic Assessment	8a	Diagnostic testing (such as PE, laboratory testing, imaging, surveys)	Page 2, Case presentation (eosinophil count, ANCA serology, chest CT, external auditory canal biopsy, immunohistochemistry CD31)
	8b	Diagnostic challenges (such as access to testing, financial, or cultural)	Page 2-3, Discussion (ANCA-negative, diagnosis challenging, histopathology essential)
	8c	Diagnosis (including other diagnoses considered)	Page 2, Case presentation (final diagnosis: ANCA-negative EGPA)
	8d	Prognosis (such as staging in oncology) where applicable	Not applicable (prognosis not specifically staged)
Therapeutic Intervention	9a	Types of therapeutic intervention (such as pharmacologic, surgical, preventive, self-care)	Page 3, Discussion (systemic corticosteroids)
	9b	Administration of therapeutic intervention (such as dosage, strength, duration)	Not specified in the manus Cript
Follow-up and Outcomes			

9c Changes in therapeutic intervention (with rationale) .... Not applicable

10a Clinician and patient-assessed outcomes (if available).......... Page 3, Abstract - Case presentation (symptoms improved after corticosteroid therapy)

10b Important follow-up diagnostic and other test results.......... Not reported in the manuscript

10c Intervention adherence and tolerability (How was this assessed?).......... Not reported in the manuscript

10d Adverse and unanticipated events.......... Not reported in the manuscript

Discussion

11a A scientific discussion of the strengths AND limitations associated with this case report.......... Page 2-3, Discussion (strengths: biopsy diagnosis; limitations: single case, limited follow-up)

11b Discussion of the relevant medical literature **with references**.......... Page 2-3, Discussion (references 1-22)

11c The scientific rationale for any conclusions (including assessment of possible causes).......... Page 3, Discussion (eosinophil degranulation, T-cell activation, vasculitis mechanism)

11d The primary "take-away" lessons of this case report (without references) in a one paragraph conclusion.......... Page 3, Discussion final paragraph

Patient Perspective

12 The patient should share their perspective in one to two paragraphs on the treatment(s) they received . . . . Not applicable (patient perspective not included)

Informed Consent

13 Did the patient give informed consent? Please provide if requested . . . . Page 3, Declarations - Consent for publication (Yes)
